# Effect of total knee arthroplasty for valgus knee correction on clinical outcome and patellar position

**DOI:** 10.1007/s00264-023-05689-x

**Published:** 2023-01-16

**Authors:** Liang Zhou, Xuening Dai, Zhongyuan Zhou, Qian Kong, Guoqing Duan, Yuanmin Zhang

**Affiliations:** 1 Department of Clinical, Jining Medical University, Jining, Shandong China; 2 Department of Operating Room, Affiliated Hospital of Jining Medical University, Jining, Shandong China; 3 Department of Sports Injury and Arthroscopy, Tianjin Hospital, Tianjin University, Hexi District, Tianjin, China; 4 Department of Orthopedics and Joints, Affiliated Hospital of Jining Medical University, Jining, Shandong China

**Keywords:** Valgus, Total knee arthroplasty, Lower limb, Patella

## Abstract

**Purpose:**

The purpose was to investigate the effect of different degrees of valgus deformity correction on patellar position and clinical outcome in patients with valgus knees after total knee arthroplasty (TKA).

**Methods:**

We retrospectively analyzed and followed 118 patients with valgus knees. Based on the post-operative hip–knee–ankle (HKA), patients were divided into three groups: neutral (±3°), mild (3–6°), and severe (> 6°). Western Ontario and McMaster Universities Osteoarthritis Index (WOMAC), range of motion (ROM), and Knee Society Score (KSS) were used to evaluate post-operative clinical efficacy. Also, the patellar tilt angle (*ε*-angle), congruence angle (*θ*-angle), and Insall–Salvati index (ISI) were used to represent the patellar position. Post-operative observation indicators included HKA, angle of the femur (*α*-angle), tibial angle (*β*-angle), femoral component flexion angle (*γ*-angle), and tibial component posterior slope angle (*δ*-angle).

**Results:**

All patients showed significant improvements in HKA, ROM, WOMAC, and KSS after operation (*P* < 0.001). Regarding patellar position, the ISI values decreased to varying degrees (*P* < 0.05). The patellar tilt angle was significantly increased in the severe valgus group compared to that in the mild valgus and neutral groups (*P* < 0.001). Univariate analysis showed that the degree of post-operative residual valgus was significantly affected by WOMAC, KSS, *α*-, *ε*-, and *θ*-angles.

**Conclusion:**

Minor valgus undercorrection did not affect the short-term outcome after TKA; however, when the residual valgus angle was > 6°, the post-operative scores were significantly reduced. Inadequate valgus correction does not result in significant changes in patellar height but may increase the risk of poor patellar tracking.

## Introduction

Total knee arthroplasty (TKA) is an effective surgical method for treating end-stage knee disease [1]. The consensus is that TKA should achieve good soft tissue balance and restore the neutral position of the lower limb force line, even if the hip–knee–ankle (HKA) mechanical axis is ± 3° [2–6]. However, whether maintaining the neutral position can achieve better clinical efficacy has not been clearly determined. Some scholars believe that poor lower limb alignment after TKA will increase the contact stress between the prosthesis and may lead to early aseptic loosening of the prosthesis, and maintaining the lower limb alignment neutral position can improve the survival rate of the prosthesis [7–9]. However, other researchers have observed that retaining a certain varus or valgus after operation has no significant effect on clinical efficacy and can improve post-operative satisfaction to a certain extent [10–15]. Among patients undergoing TKA, approximately 10% have valgus deformity [16, 17]. Compared with varus knees, the pathological changes of valgus knees are more complex, often accompanied by bone defects, medial collateral ligament relaxation, etc. Additionally, the patellar position significantly influences the biomechanics of the knee joint. The height and trajectory of the patella may affect knee joint function [18]. Some patients with valgus knees have an excessive *Q* angle and poor patellar trajectory due to contracture and valgus deformity of the lateral supporting ligament. TKA is often ineffective due to the above reasons; hence, the need to increase the amount of distal femoral osteotomy and lateral soft tissue release will lead to post-operative patellar position changes, which may impact knee function.

## Materials and methods

**Fig. 1:**
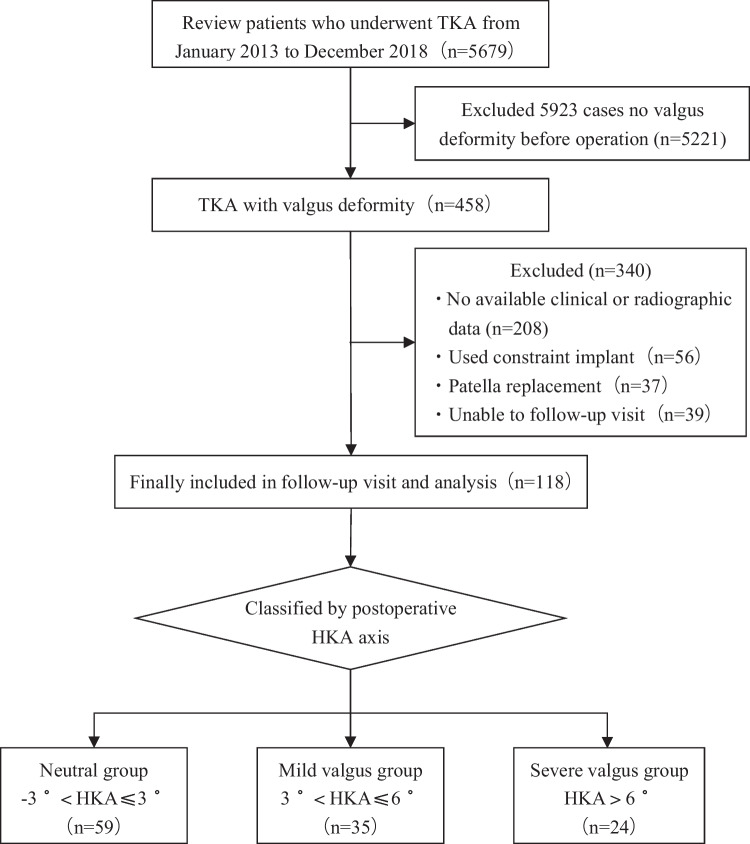
Flowchart of patients included in the study

This study retrospectively analyzed and followed 118 patients who underwent TKA for knee osteoarthritis with valgus deformity between January 2013 and December 2018 at the Department of Orthopaedics, Affiliated Hospital of XXX Medical University. Figure 1 presents the screening process. Among all patients, 88 were diagnosed with primary osteoarthritis and 30 with rheumatoid arthritis. The surgical indications for all patients were pain, limited activity, and a serious impact on daily life. The inclusion criteria of this study were as follows: (1) Patients with knee osteoarthritis combined with knee valgus deformity, (2) the use of knee prosthesis as a posterior stable prosthesis, and (3) there were no medial or lateral collateral ligament injuries. The exclusion criteria were as follows: (1) incomplete pre-operative and post-operative imaging data; (2) pre-operative ipsilateral knee with no history of trauma, infection, or surgery; (3) patients who could not follow the doctor's advice for rehabilitation exercises for various reasons, and (4) patients undergoing patellar replacement.

Based on the post-operative HKA alignment, the patients were divided into three groups: neutral (− 3°–3°), mild (3–6°), and severe (> 6°). The general patient information is shown in Table 1. This study was approved by the Ethics Committee of the Affiliated Hospital of Jining Medical University. We obtained informed consent from all participants.

**Table 1:** Patient general information

Group	Head	Gender		Age (years)	BMI (kg/m^2^)	Affected limbs		Diagnosis		Follow-up duration(months)
		Male	Female			Left	Right	OA	RA	
Neutral group	59	8	51	64.24 ± 8.33	25.55 ± 3.70	20	39	44	15	55.19 ± 7.33
Mild group	35	6	29	63.26 ± 8.17	26.10 ± 3.96	13	22	24	11	56.57 ± 7.92
Severe group	24	4	20	63.63 ± 8.72	25.68 ± 4.36	8	16	20	4	53.79 ± 8.30
*X*^2^/*F*/*H*	-	0.265		0.159	0.455	0.129		1.636		2.169
*P*	-	0.876		0.853^#^	0.796^&^	0.938		0.441		0.338^&^

- represents no data; count data using chi-square test; # represents a single-factor analysis of variance; & represents the Kruskal–Wallis test

### Surgical techniques and management

After the patient entered the operating room, the surgeon, anesthesiologist, and nurse checked the patient’s information and the surgical site. The patient was placed in the supine position, and general anaesthesia combined with nerve block anesthesia was performed. After successful anaesthetization, the pneumatic tourniquet was tied (the pressure of the pneumatic tourniquet during the operation was 300 mmHg), the skin was routinely disinfected, a sterile towel was spread, and the skin membrane was covered to prevent infection. For the median knee incision, the medial patellar approach was used to explore the joint surface damage. Hyperplasia of synovial tissue resection, clean-up of osteophytes, protection of the tissue around the knee joint, conventional osteotomy, and tibial plateau hardening zone drilling of multiple holes were performed. In addition, patellar repair and patellar periphery denervation were performed. A cocktail of ropivacaine 100 mg, dexamethasone 5 mg, and morphine 2 mg, diluted to 40 ml with normal saline, was injected around the knee joint; bone cement was applied, and an appropriate prosthesis was placed (Zimmer ® NexGen LPS-flex, USA or Biomet ® Vanguard PS, USA) according to the size measured after osteotomy. Haemostasis, a large number of saline rinses, and layer-by-layer sutures were performed. None of the patients underwent patellar replacement. If there was lateral soft tissue tension after osteotomy, a 50-ml needle was used to make a pie-crusting method to release soft tissue, and if there was a bone defect, filling was done with a part of the screw and bone cement. All patients began active and passive activities on the second day after the operation. All operations were performed by two experienced surgeons.

### Clinical and radiographic assessment

**Fig. 2:**
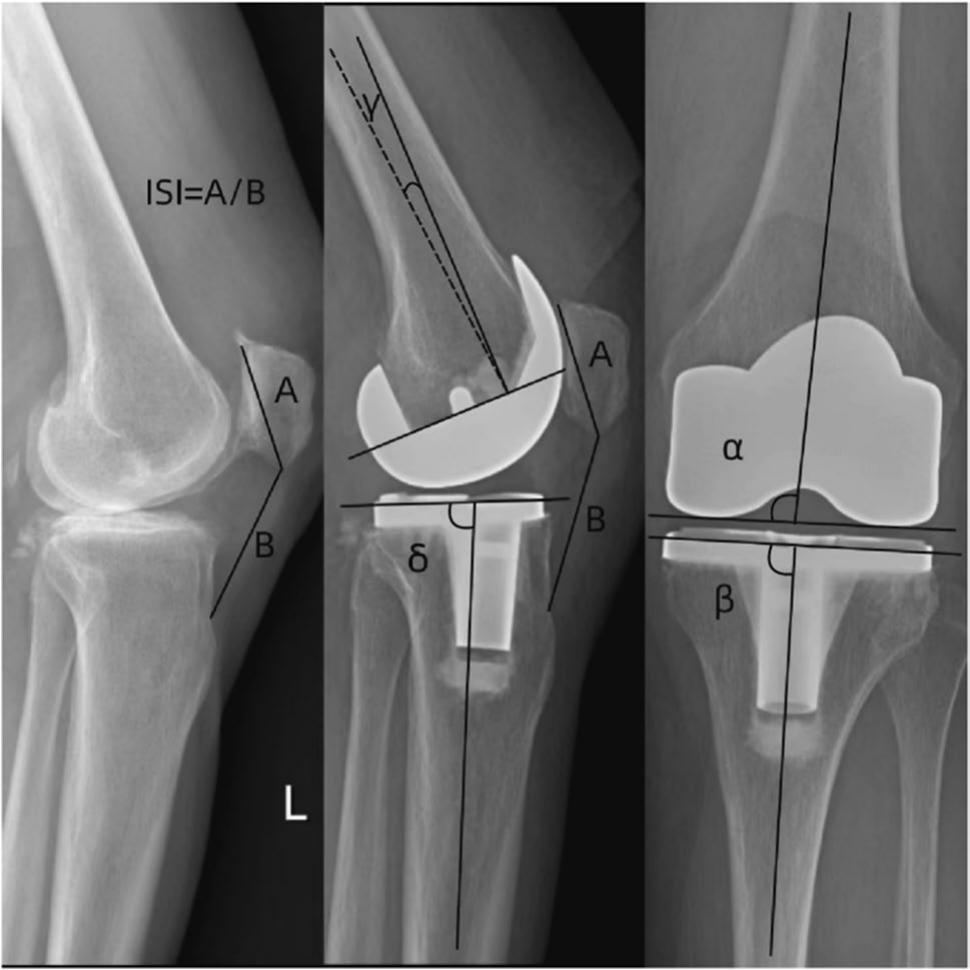
The ISI, *α*-angle, *β*-angle, *γ*-angle, and *ε*-angle were measured on the pre-operative knee lateral X-ray and post-operative knee anteroposterior X-ray and lateral X-ray

**Fig. 3:**
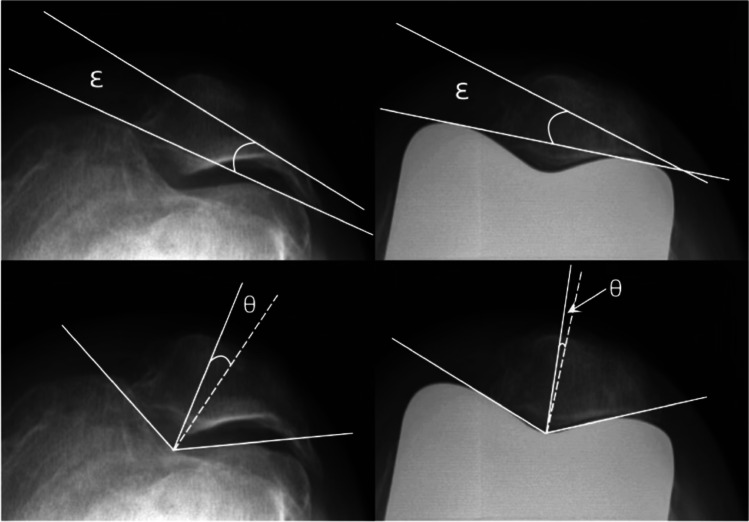
The *ε*-angle and *θ*-angle were measured on the pre-operative and post-operative patellar axial films. Note: the solid line represents the bisector of the angle of the bone groove, and the dotted line shows the connection between the lowest point of the intercondylar groove and the joint edge of the patella

Clinical assessments included knee range of motion (ROM), Western Ontario and McMaster University Osteoarthritis Index (WOMAC) scores, and Knee Society Scores (KSS) before operation and at the last follow-up. Radiographic measurements included the HKA, Insall–Salvati Index (ISI), patellar tilt angle (*ε*-angle), and congruence angle (*θ*-angle). The above indices were measured on pre-operative and final follow-up X-ray films, including anteroposterior, lateral, and Merchant positions of the knee joint when standing and loading. HKA (positive value indicates valgus) was measured on the pre-operative and post-operative full-length radiographs of the lower extremities. To study the position of the knee prosthesis on the coronal and sagittal planes, the femoral angle (*α*-angle), tibiofemoral angle (*β*-angle), femoral prosthesis flexion angle (*γ*-angle), and tibial prosthesis posterior slope angle (*δ*-angle) were measured by standing straight knee and lateral radiographs. The femoral angle is the medial angle between the femoral anatomical axis and the tangent of the femoral prosthesis. The tibiofemoral angle is the medial angle between the tibial anatomic axis and the articular surface tangent of the tibial prosthesis. The flexion angle is the angle between the anatomical axis of the femur and the vertical line of the femoral prosthesis. The slope angle is the angle between the tibial and tibial anatomical axes (Fig. 2). To determine the relative position of the patella before and after surgery, the ISI, patellar tilt angle, and congruence angle were measured using lateral knee radiographs (flexion 30–60°) and Merchant view. The patellar tilt angle is the angle between the maximum transverse diameter of the patella and the line connecting the highest point of the femoral prosthesis. The congruence angle is the angle between the bisector of the trochlear angle of the femoral prosthesis and the line connecting the trochlear roof and the inferior pole of the patella. The positive value of the patellar tilt angle indicated lateral displacement of the patella (Fig. 3). A patellar tilt angle > 10° was defined as abnormal [19], and a congruence angle > 16° was defined as abnormal [20]. The above angles were measured using a picture archiving and communication system.

The imaging values were measured by two experienced orthopedists. Each value was measured twice. If the results were inconsistent, the average was considered. Finally, the pre-operative and post-operative imaging data of the three groups were compared.

### Statistical analysis

Data analysis was done using Statistical Product Service Solutions 26.0 (SPSS 26.0). The measurement data were described by $\overline{x}$ ± *s*, and the count data were described by frequency (percentage). Univariate analysis, Kruskal–Wallis test, and chi-square test were used for comparisons between groups. Indicators with statistical significance between groups were corrected using Bonferroni’s correction. The effects of post-operative HKA on the patellar tilt angle, fitness angle, KSS total score, WOMAC total score, and ISI were analyzed using linear regression analysis. The paired *t*-test and signed-rank sum test were used to compare the differences before and after surgery in the groups. Statistical significance was set at *P* < 0.05.

## Results

There was no significant difference in the pre-operative data of the three groups, except for HKA (Table 2). Comparing the pre-operative and post-operative clinical data of the three groups of patients showed that the post-operative ROM, WOMAC score, KSS, and HKA alignment significantly improved (*P* < 0.001). ISI showed a downward trend (*P* < 0.05). The post-operative *ε*-angle was significantly increased compared to before operation (*P* < 0.001), and the post-operative *θ*-angle was not significantly changed compared to before operation (Table 3).

**Table 2:** Pre-operative data of patients

Group	ROM (°)	HKA (°)	ISI	WOMAC				KSS			*ε*-angle (°)	*θ*-angle(°)
				Pain	Stiff	Daily	Total	Knee score	Functional score	Total		
Neutral group (*n* = 59)	95.85 ± 22.90	9.22 ± 4.08	1.01 ± 0.10	15.36 ± 1.51	6.15 ± 1.01	38.14 ± 3.21	59.64 ± 4.57	56.66 ± 6.63	52.63 ± 2.52	109.29 ± 7.16	6.19 ± 1.72	7.40 ± 4.56
Mild group (*n* = 35	99.54 ± 20.29	8.71 ± 5.60	1.01 ± 0.12	15.69 ± 1.91	5.69 ± 1.30	37.11 ± 2.31	58.49 ± 4.64	55.11 ± 8.11	51.57 ± 4.97	106.69 ± 9.70	6.62 ± 1.99	8.79 ± 5.81
Severe group (*n* = 24)	95.17 ± 20.14	13.74 ± 5.89	1.03 ± 0.18	16.42 ± 2.60	6.29 ± 1.04	38.67 ± 3.66	61.38 ± 6.37	54.79 ± 3.85	50.63 ± 4.50	105.42 ± 5.98	7.01 ± 1.06	10.70 ± 7.17
*H*/*F*	0.749	14.793	0.171	2.517	4.900	3.845	2.375	1.795	3.072	4.346	5.595	4.181
*P*	0.688^&^	*0.001*^*&*^	0.918^&^	0.284^&^	0.086^&^	0.146^&^	0.098^#^	0.408^&^	0.215^&^	0.114^&^	0.061^&^	0.124^&^

# represents single-factor analysis of variance, & represents the Kruskal–Wallis test

**Table 3:** Comparison of post-operative clinical data between the groups

Group	Neutral group (*n* = 59)				Mild group (*n* = 35)				Severe group (*n* = 24)			
	Pre-operative	Post-operative	*Z*	*P* value	Pre-operative	Post-operative	*Z*	*P* value	Pre-operative	Post-operative	*Z*	*P* value
ROM(°)	95.85 ± 22.90	112.80 ± 13.84	− 6.526	*< 0.001*	99.54 ± 20.29	117.00 ± 18.91	− 4.432	*< 0.001*	95.17 ± 20.14	110.63 ± 10.35	− 3.833	*< 0.001*
HKA(°)	9.22 ± 4.08	0.85 ± 1.61	− 6.680	*< 0.001*	8.71 ± 5.60	4.58 ± 0.98	− 4.046	*< 0.001*	− 13.74 ± 5.89	− 8.36 ± 1.97	− 3.415	*0.001*
ISI	1.01 ± 0.10	0.98 ± 0.10	− 2.094	*0.036*	1.01 ± 0.12	0.98 ± 0.14	− 1.979	*0.048*	1.03 ± 0.18	0.99 ± 0.18	2.482	*0.021*^*ƚ*^
WOMAC-pain	15.36 ± 1.51	3.61 ± 0.97	− 6.708	*< 0.001*	15.69 ± 1.91	3.74 ± 0.74	− 5.176	*< 0.001*	16.42 ± 2.60	4.92 ± 1.77	− 4.294	*< 0.001*
WOMAC-stiff	6.15 ± 1.01	3.03 ± 0.77	− 6.630	*< 0.001*	5.69 ± 1.30	3.26 ± 0.56	− 5.050	*< 0.001*	6.29 ± 1.04	4.04 ± 0.91	− 4.241	*< 0.001*
WOMAC-daily	38.14 ± 3.21	3.76 ± 0.70	− 6.695	*< 0.001*	37.11 ± 2.31	4.00 ± 0.77	− 5.191	*< 0.001*	38.67 ± 3.66	4.33 ± 1.13	− 4.293	*< 0.001*
WOMAC-total	59.64 ± 4.57	10.41 ± 1.61	− 74.903	*< 0.001*^*ƚ*^	58.49 ± 4.64	11.00 ± 1.44	− 5.164	*< 0.001*	61.38 ± 6.37	13.29 ± 2.73	− 35.067	*< 0.001*^*ƚ*^
KSS-knee score	56.66 ± 6.63	85.93 ± 3.15	− 6.708	*< 0.001*	55.11 ± 8.11	84.46 ± 3.17	− 5.163	*< 0.001*	54.79 ± 3.85	77.33 ± 3.50	− 4.298	*< 0.001*
KSS-functional score	52.63 ± 2.52	76.61 ± 4.09	− 6.764	*< 0.001*	51.57 ± 4.97	74.86 ± 3.53	− 5.205	*< 0.001*	50.63 ± 4.50	68.96 ± 6.25	− 4.328	*< 0.001*
KSS-total	109.29 ± 7.16	162.54 ± 5.28	− 6.698	*<0.001*	106.69 ± 9.70	159.31 ± 5.00	− 5.162	*< 0.001*	105.42 ± 5.98	146.29 ± 7.03	27.048	*< 0.001*^*ƚ*^
*ε*-angle(°)	6.19 ± 1.72	7.30 ± 1.45	4.684	*< 0.001*^*ƚ*^	6.62 ± 1.99	8.61 ± 2.78	− 3.612	*< 0.001*	7.01 ± 1.06	13.82 ± 3.66	8.911	*< 0.001*^*ƚ*^
θ-angle(°)	7.40 ± 4.56	7.40 ± 4.21	− 0.429	0.668	8.79 ± 5.81	8.68 ± 5.30	− 0.060	0.952	10.70 ± 7.17	10.26 ± 6.20	− 1.297	0.208^*ƚ*^

*t* means paired *t*-test, otherwise paired-rank sum test

The comparison of post-operative clinical data among the three groups showed that the KSS-knee score and KSS-total of the mild group were significantly lower than those of the neutral group, and the *α*-angle was significantly increased (*P* < 0.001). Compared with the neutral group, the severe group showed significant differences in more aspects, which were manifested in the significant reduction of WOMAC and KSS, except WOMAC-daily, and a significant increase in the *α*-angle and *ε*-angle. In addition, the ROM and WOMAC scores, except WOMAC-daily and KSS scores, were significantly lower, and the *α*-angle and *ε*-angle were significantly increased in the severe group than in the mild group (*P* < 0.001) (Table 4).

**Table 4:** Comparison of post-operative clinical data between the groups

Item	Neutral group (*n* = 59)	Mild group (*n* = 35)	Severe group (*n* = 24)	*H*/*F*	*P* value
ROM	112.80 ± 13.84	117.00 ± 18.91	*110.63 ± 10.35*^*b*^	6.998	0.030^&^
ISI	0.98 ± 0.10	0.98 ± 0.14	0.99 ± 0.18	0.236	0.889^&^
WOMAC-pain	3.61 ± 0.97	3.74 ± 0.74	*4.92 ± 1.77*^*a*^	10.112	0.006^&^
WOMAC-stiff	3.03 ± 0.77	3.26 ± 0.56	*4.04 ± 0.91*^*ab*^	22.337	*< 0.001*^*&*^
WOMAC-daily	3.76 ± 0.70	4.00 ± 0.77	4.33 ± 1.13	5.346	0.069^&^
WOMAC-total	10.41 ± 1.61	11.00 ± 1.44	*13.29 ± 2.73*^*ab*^	20.355	*< 0.001*^*&*^
KSS-knee score	85.93 ± 3.15	*83.11 ± 4.20*^*a*^	*77.33 ± 3.50*^*ab*^	53.080	*< 0.001*^*&*^
KSS-functional score	76.61 ± 4.09	74.43 ± 3.16	*68.96 ± 6.25*^*ab*^	30.171	*< 0.001*^*&*^
KSS-total	162.54 ± 5.28	*157.54 ± 5.64*^*a*^	*146.29 ± 7.03*^*ab*^	56.578	*< 0.001*^*&*^
*α*-angle	95.32± 1.08	*96.72 ± 0.86*^*a*^	*98.68 ± 1.25*^*ab*^	87.850	*< 0.001*^*#*^
*β*-angle	90.10 ± 0.83	90.43 ± 0.76	90.26 ± 0.82	1.950	0.147^#^
*γ*-angle	1.92 ± 0.53	1.94 ± 0.51	2.13 ± 0.57	3.715	0.156^&^
*δ*-angle	85.30 ± 0.68	85.36 ± 0.71	85.29 ± 0.70	0.115	0.892^#^
*ε*-angle	7.30 ± 1.45	*8.61 ± 2.78*^*a*^	*13.82 ± 3.66*^*ab*^	52.626	*<0.001*^*&*^
*θ*-angle	7.40 ± 4.21	8.68 ± 5.30	10.26 ± 6.20	5.055	0.080^&^

# represents single-factor analysis of variance; & represents the Kruskal–Wallis test

^a^Statistically significant compared with the neutral position group (*P* < 0.05)

^b^Statistically significant compared with the mild valgus group (*P* < 0.05)

The effect of post-operative HKA on the post-operative data was analyzed using linear regression. The results showed that post-operative HKA had a significant effect on WOMAC-pain, WOMAC-stiff, WOMAC-total, KSS-knee, KSS-function, KSS-total, *α*-angle, *ε*-angle, and *θ*-angle (*P* < 0.001) (Table 5).

**Table 5:** Effects of post-operative HKA on other post-operative indicators

Item	Unstandardized coefficients		*Β*	*T*	*P* value
	*B*	Root mean squared error			
ROM	− 0.154	0.419	− 0.034	− 0.368	0.714
ISI	0.002	0.004	0.064	0.691	0.491
WOMAC-pain	0.161	0.031	0.438	5.241	*< 0.001*
WOMAC-stiff	0.112	0.021	0.450	5.422	*< 0.001*
WOMAC-daily	0.073	0.023	0.286	3.212	*0.002*
WOMAC-total	0.346	0.050	0.539	6.884	*< 0.001*
KSS-knee score	− 0.933	0.103	− 0.644	− 9.072	*< 0.001*
KSS-functional score	− 0.899	0.120	− 0.571	− 7.493	*< 0.001*
KSS-total	− 1.832	0.163	− 0.722	− 11.232	*< 0.001*
*α*-angle	0.422	0.025	0.842	16.787	*< 0.001*
*β*-angle	0.025	0.023	0.100	1.083	0.281
*γ*-angle	0.015	0.015	0.094	1.022	0.309
*δ*-angle	0.013	0.019	0.062	0.669	0.505
*ε*-angle	0.783	0.065	0.746	12.060	*< 0.001*
*θ*-angle	0.521	0.133	0.342	3.916	*< 0.001*

post-operative HKA is the independent variable, and the project part is the dependent variable

## Discussion

The main finding of this study is that in patients with valgus knee deformity before TKA, a slight insufficient correction after TKA of the valgus will not affect the patient’s short-term clinical efficacy; however, there may be a risk of poor patellar tracking. Furthermore, severe undercorrection will affect the recent clinical outcomes of patients, and the risk of poor patellar tracking will also increase.

Owing to the error in traditional tool measurement and the lack of fine manual operation, it is very common to have a certain degree of deformity correction after TKA [21]. Early studies have reported that poor alignment of the lower limb after TKA affects the biomechanics of the lower limbs and leads to poor clinical results [2, 22, 23]. However, approximately 25% of patients with neutral lower limb alignment after TKA have not achieved satisfactory results [24, 25]. This may be because TKA patients have severe deformities before surgery, which requires more complex osteotomy and more soft tissue release to achieve neutral alignment; however, this will also cause greater damage and may result in poor clinical outcomes [26]. Slevin et al. reported that soft tissue tension affects the neurosensory reflex, which affects post-operative outcomes and patient satisfaction [6]. This concept is similar to motion alignment, which involves maintaining normal knee kinematics and minimizing the release of soft tissue around the joint to achieve better clinical results [27]. A recent meta-analysis showed that motion alignment during TKA can achieve better clinical results and patient satisfaction than mechanical alignment [28]. In this study, similar reasons may have affected the results. The neutral position group did not show better clinical results than the mild valgus group, which may be due to the difference in pre-operative HKA (neutral group, 9.22°; mild group, 8.71°).

With the deepening of TKA research and the development of surgical techniques and prostheses in recent years, an increasing number of scholars and studies have reported that post-operative mechanical irregularity of the lower limbs is not the main cause of TKA failure [4, 29]. However, there is no definite conclusion about the relationship between lower limb alignment and knee function after TKA. Some scholars have reported that patients with mild varus correction can achieve better or similar clinical results than those with neutral lower limb alignment after surgery [11, 12, 15, 30–33]. Moreover, the pathological process of TKA in patients with valgus knees is more complex than that in patients with varus knees, and the operation is more difficult. There is no consensus on the surgical approach, soft tissue release, or prosthesis selection [34]. Some scholars have observed that the results of mild undercorrection in patients with valgus knees after operation have achieved almost the same score as the results of neutral position [15, 35]; however, excessive undercorrection has achieved a poor score [15]. Similar results were obtained in this study. There was no significant difference in the score between the mild valgus undercorrection (3° < HKA < 6°) and neutral groups, while the score was significantly reduced when the valgus residue was excessive (HKA > 6°).

Studies have shown that poor patellar tracking after TKA can lead to post-operative pain and decreased patient satisfaction [36–38]. Compared with varus deformity in patients with post-operative residual varus, valgus knee patients with post-operative residual valgus have a worse clinical effect when the incidence of patellar maltracking is higher [11, 12]. Slevin et al. demonstrated that the degree of valgus after TKA is the most relevant factor for patellar maltracking occurrence [39]. At the same time, some scholars have observed that the poor patellar trajectory after TKA in patients with valgus knees is related to the release of soft tissue and an increase in the *Q* angle [22]. In this study, the post-operative patellar tilt angle showed an increasing trend with the lack of valgus correction, and the comparison between the groups was statistically significant, which is consistent with previous research findings. The patellar trajectory is affected by many factors, including the lower limb force line, the height of the joint line, and the position of the prosthesis. Moreover, because CT is not used as a routine post-operative examination, it is impossible to fully evaluate the effect of the prosthesis position on the patellar trajectory.

The position of the patella has a great influence on the biomechanics of the knee joint, and reduction in the position of the patella after TKA is a common post-operative complication. Abnormal patellar height may affect the knee joint [18]. sTKA for severe valgus deformities may increase the thickness of the cut bone and soft tissue release, leading to changes in patellar height and affecting knee function. Previous studies on residual valgus after valgus knee TKA did not include an index of patellar height, which may have led to the neglect of this influencing factor. Based on previous studies, this study included an index of patellar height, which can more comprehensively analyze the relationship between residual valgus and knee function after TKA of valgus knees. Reportedly, the ISI is a reliable basis for evaluating patellar height [40]; therefore, this study used the ISI to represent the relative height of the patella before and after operation. The results showed that there was no significant difference in ISI between the three groups before and after operation, and there was no significant difference between the pre-operative and post-operative ISI groups. Linear regression analysis showed that post-operative HKA had no significant effect on the ISI. There was no significant decrease in the post-operative patellar height, which may be related to the small sample size of this study.

This study had some limitations. Firstly, the follow-up period was short, and it is difficult to prove whether the correction of valgus knees after TKA is related to the durability of the prosthesis and the success of the operation. Secondly, this study was a single-center retrospective study, according to the post-operative HKA group, with no random sampling, making the comparison between groups unreliable. Finally, the lower limb HKA was measured based on the full-length X-ray of the lower limb, which was numerically less reliable than the three-dimensional computed tomography reconstruction. In the later stages, we will continue to follow up on these patients to obtain long-term research results.

In conclusion, the degree of correction of lower limb alignment after TKA is associated with the clinical effect and will affect the position of the patella; however, a slight insufficient correction will hardly affect short-term clinical efficacy. Moreover, excessive post-operative residual valgus (> 6°) will affect short-term clinical efficacy, and an insufficient correction may increase the risk of poor patellar tracking. Finally, although the height of the patella decreased to different degrees after TKA, the degree of correction did not affect the degree of height reduction.

## Data Availability

The datasets used or analyzed during the current study are available from the corresponding author upon reasonable request.
